# Differential Effects of Chronic Alcohol Consumption on Cortical and Subcortical Brain Volume in Adolescent Nonhuman Primates

**DOI:** 10.1523/ENEURO.0353-19.2019

**Published:** 2019-09-30

**Authors:** Rosalind S.E. Carney

## Abstract

**Highlighted Research Paper:** Chronic Alcohol Drinking Slows Brain Development in Adolescent and Young Adult Nonhuman Primates, by Tatiana A. Shnitko, Zheng Liu, Xiaojie Wang, Kathleen A. Grant, and Christopher D. Kroenke.

Alcohol is the predominant substance of abuse experimented with by adolescents in the United States. The 2015 National Survey on Drug Use and Health reported that 7.7 million individuals aged 12–20 had consumed alcohol in the month preceding survey (Substance Abuse and Mental Health Services Administration, 2015). Compared to adults, adolescents are less resistant to the intoxicating effects of alcohol, and although adolescents tend to drink on fewer occasions than adults, they are more prone to binge drinking (defined as the consumption of five or more drinks on the same occasion for boys or four or more drinks on the same occasion for girls on at least 1 d in the past 30 d; National Institute on Alcohol Abuse and Alcoholism, 2006). Magnetic resonance imaging (MRI) studies have searched for the neural basis of the increased susceptibility of adolescents to the rewarding effects of alcohol. For example, increased activation of the nucleus accumbens during decision making that involved risk and reward was observed in alcohol-naive study participants who progressed to binge drinking earlier than their minimal alcohol use counterparts (Morales et al., 2018). As brain maturation continues into the third decade of life, MRI studies have also addressed the adverse effects of alcohol consumption on brain development. Normotypic changes in brain volume (V_B_) during adolescence include a decrease in cortical gray matter and an increase in white matter due to synaptic pruning and myelination, respectively (Giedd et al., 1999; Lebel et al., 2008; Sullivan et al., 2011; Yeatman et al., 2014; Levman et al., 2017; Narvacan et al., 2017). Heavy alcohol consumption also results in cortical gray matter reduction but attenuates white matter growth (Squeglia et al., 2014). During normotypic development, specific structures exhibit age-related, nonlinear growth trajectories (Sowell et al., 2004; Sullivan et al., 2011; Dennison et al., 2013; Barnea-Goraly et al., 2014; Yeatman et al., 2014; Bernard et al., 2015). In a prior study, Professor Christopher Kroenke and colleagues (Oregon Health and Science University, Portland, WA) found that chronic alcohol consumption by adult nonhuman primates (NHPs) led to a decrease in cortical volume; the most cortical gray shrinkage occurred in NHPs that had the highest levels of alcohol consumption (Kroenke et al., 2014). In their *eNeuro* publication, Shnitko et al., 2019 examined how alcohol consumption affects the growth trajectories of cortical and subcortical brain structures of NHPs from adolescence to early adulthood.

The NHPs used in the study were male (*n* = 58) and female (*n* = 13) rhesus monkeys used in ongoing research projects by a consortium of researchers at the Oregon National Primate Research Center. Data and analyses from these studies are available from the Monkey Alcohol Tissue Research Resource database (MATRR; www.matrr.com). NHPs were studied in cohorts of 8–12, and most were 4.5–5.5 years old at the start of the study. Weight was measured weekly so that alcohol intake could be accurately expressed as grams per kilogram of contemporary body weight. Alcohol was delivered via a computerized panel inserted into the housing cage that contained drinking spouts which could be rendered inoperable when needed. The authors used a schedule-induced polydipsia protocol in which the NHPs were induced to orally self-administer escalating doses of 4% ethanol solution equivalent to two, four, and six drinks per day and then allowed 22 h/d to choose to drink as much alcohol as they wanted with water always available. This stage of “open access” to ethanol was 7 d/week over 12 months. The range of resulting average daily alcohol consumptions mimics the range of consumption in humans, with ∼50% of the monkeys categorized as heavy drinkers (HDs; >20% of days consuming over 12 drinks) which were compared with low drinkers and binge drinkers, which were combined as non-HDs.

The NHPs underwent MRI sessions at three time points during the study: the baseline MRI (MRI_1_) was performed before alcohol self-administration induction sessions, and MRI_2_ and MRI_3_ were performed after six months and one year, respectively, of open-access self-administration of alcohol. Linear regression analysis was used to access changes in V_B_ in 10 regions of interest, across the three MRI time points.

Shnitko and colleagues observed that brain growth continued into young adulthood, corresponding to ∼7.5 years of age in the NHP model (approximately mid-twenties for humans). Initially, the authors were surprised to observe that the brain grows over this period, not only in control animals but also in HDs. During late adolescence and early adulthood, V_B_ increased by 1 ml/1.87 years in control animals. However, investigation of the effect of chronic alcohol exposure revealed that brain growth was reduced by 0.25 ml/year per 1 g/kg of daily ethanol (Fig. 1). The authors also conducted analyses of brain subregions. They were not able to detect age-related volume changes in the caudate nucleus, hippocampus, putamen, cortex, or amygdala. In contrast, an age-related volume increase from baseline was observed in the brainstem (5%/year), globus pallidus (3.2%/year), and the cerebellum (1.6%/year) during the 3.9–7.9 years of age time frame. However, the most significant impact of chronic alcohol consumption was observed for the white matter and thalamus. In CTRs, white matter volume increased at a rate of 0.6 ml/year; white matter growth was attenuated by 0.25 ml/year in HDs (Fig. 2). In CTRs, thalamic volume increased at a rate of 0.06 ml/year, but its growth was attenuated by 0.02 ml/year in HDs (Fig. 3). These results indicate that sensory and limbic, rather than cortical, structures are particularly susceptible to the adverse effects of alcohol during the time frame that corresponds to adolescence in humans.

**Figure 1.**
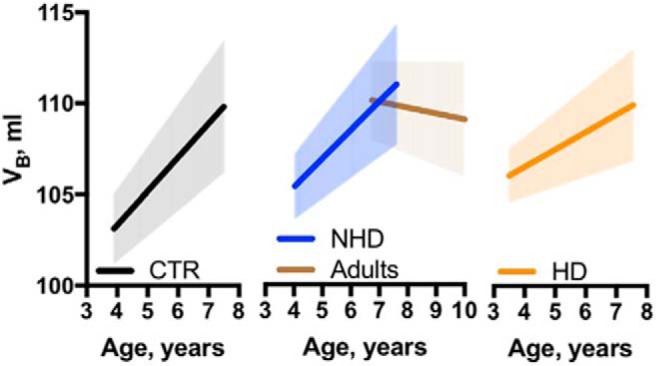
Age-dependent brain growth in NHPs. Average V_B_-by-age linear regression estimated for CTRs, NHDs, and HDs with 95% confidence interval depicted by the shaded area around the line (Adapted from Figure 2 in Shnitko et al., 2019).

**Figure 2.**
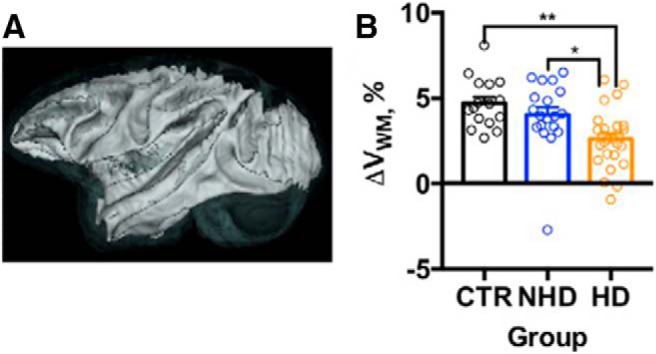
Heavy ethanol intake reduces the rate of the white matter growth in NHP brain. ***A***, 3D representation of the cortical white matter in the brain. ***B***, The estimated rate of white matter growth (ΔV_WM_, %) in the CTRs, NHDs, and HDs. The shadows above and below the regression lines depict the 95% confidence interval. The dots represent change in the volume measured in individual NHPs. Asterisks show the results of Bonferroni *post hoc* test, where *p* values adjusted for the multiple comparisons were **p < 0.01 and *p < 0.05 (Adapted from Figure 4 in Shnitko et al., 2019).

**Figure 3.**
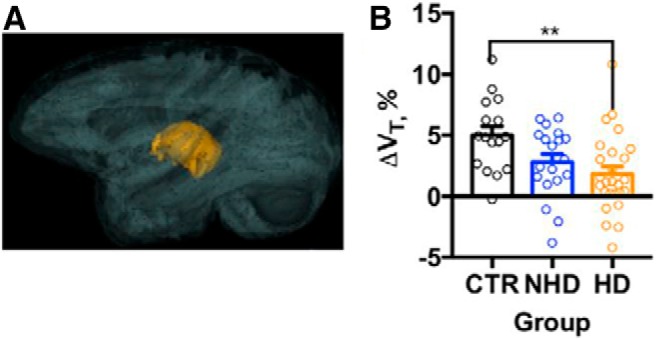
Ethanol drinking attenuates thalamic growth in adolescent/early adult NHPs. ***A***, 3D representation of the thalamus in the brain. ***B***, The estimated rate of the thalamic growth (ΔV_T_, %) in the CTRs, NHDs, and HDs. The shadows above and below the regression lines depict 95% confidence interval. The dots represent change in the volume measured in individual monkeys. Asterisks show the results of a Bonferroni *post hoc* test, where *p* values adjusted for the multiple comparisons were **p < 0.01 (Adapted from Figure 3 in Shnitko et al., 2019).

This *eNeuro* publication by Shnitko and colleagues provides detailed knowledge on normotypic growth rates in the NHP brain that coincide with the timeframe of heightened sensitivity to toxic insults caused by several substances of abuse. These data are useful for translational studies that try to distinguish factors that increase susceptibility to participate in maladaptive behaviors from the adverse effects that may result from such behaviors. Imaging studies in rodents have shown that chronic alcohol exposure reduces cortical thickness and attenuates brain growth (Pfefferbaum et al., 2006; Vetreno et al., 2017). Whereas rodent models are an invaluable tool to examine the cellular and molecular effects of alcohol exposure, their rapid brain development precludes the use of these models to recapitulate human adolescence in a reliable manner. While cross-sectional and longitudinal studies in humans also provide important insights into the adverse effects of alcohol on brain development, they contain some inherent caveats. For example, the accuracy of past alcohol consumption reporting may vary between participants. Moreover, physiologically relevant factors such as diet and alcohol intake per kilogram of contemporary body weight are difficult to control. As youths may first experiment with alcohol from as early as nine years of age (National Institute on Alcohol Abuse and Alcoholism, 2006), researchers are increasing efforts to ensure the incorporation of alcohol-naive youths into longitudinal studies that will capture youths who may experiment with alcohol at a later stage (Jones et al., 2018). For example, the ongoing National Consortium on Alcohol and Neurodevelopment (N-CANDA) studies alcohol-naive youths (Brown et al., 2015). The importance of such baseline imaging before exposure to substances of abuse was stressed in a prior study that showed significant changes in functional connectivity occur in the rodent brain following short periods of exposure to or abstinence from cocaine (Orsini et al., 2018).

Although structural changes do not necessarily exhibit a positive correlation with functional changes, it will be interesting to compare the observations from this *eNeuro* publication with a prior study that used functional MRI to determine brain network connectivity patterns during adolescence in humans (Kolskår et al., 2018). This comparison may help refine which brain structures and time points should be a main focus for the development of interventions that could mitigate the susceptibility of youths to initiate maladaptive behaviors. Ongoing and future studies by the consortium of researchers at Oregon Health and Science University aim to determine the developmental processes that underlie changes in V_B_ during adolescence in the NHP and how alcohol exposure may interfere with these processes. The differential effects of chronic alcohol exposure on cortical gray matter volume in the adolescent and adult brain suggest that treatment approaches to mitigate the adverse effects of alcohol should consider age as an important factor. The consortium researchers are also examining cognitive defects and epigenetic changes that may result from chronic alcohol exposure in NHPs.
